# Lipids as a key element of insect defense systems

**DOI:** 10.3389/fgene.2023.1183659

**Published:** 2023-06-09

**Authors:** Anna Katarzyna Wrońska, Agata Kaczmarek, Mieczysława Irena Boguś, Anna Kuna

**Affiliations:** ^1^ Museum and Institute of Zoology, Polish Academy of Science, Warszawa, Poland; ^2^ Witold Stefański Institute of Parasitology, Polish Academy of Sciences, Warsaw, Poland; ^3^ Independent Researcher, Warsaw, Poland

**Keywords:** fungal infection, lipid, insect defense mechanisms, hemocyte, cuticle, arachiadonic acid metabolites

## Abstract

The relationship between insect pathogenic fungi and their insect hosts is a classic example of a co-evolutionary arms race between pathogen and target host: parasites evolve towards mechanisms that increase their advantage over the host, and the host increasingly strengthens its defenses. The present review summarizes the literature data describing the direct and indirect role of lipids as an important defense mechanism during fungal infection. Insect defense mechanisms comprise anatomical and physiological barriers, and cellular and humoral response mechanisms. The entomopathogenic fungi have the unique ability to digest the insect cuticle by producing hydrolytic enzymes with chitin-, lipo- and proteolytic activity; besides the oral tract, cuticle pays the way for fungal entry within the host. The key factor in insect resistance to fungal infection is the presence of certain types of lipids (free fatty acids, waxes or hydrocarbons) which can promote or inhibit fungal attachment to cuticle, and might also have antifungal activity. Lipids are considered as an important source of energy, and as triglycerides are stored in the fat body, a structure analogous to the liver and adipose tissue in vertebrates. In addition, the fat body plays a key role in innate humoral immunity by producing a range of bactericidal proteins and polypeptides, one of which is lysozyme. Energy derived from lipid metabolism is used by hemocytes to migrate to the site of fungal infection, and for phagocytosis, nodulation and encapsulation. One polyunsaturated fatty acid, arachidonic acid, is used in the synthesis of eicosanoids, which play several crucial roles in insect physiology and immunology. Apolipoprotein III is important compound with antifungal activity, which can modulate insect cellular response and is considered as important signal molecule.

## 1 Introduction

After the Nobel Prize in Medicine was awarded to Bloch and Lynen for their contributions to the study of lipid metabolism (Kennedy and Westheimer, 1964), the function, metabolism and biochemistry of this group of compounds have received considerable attention. Although most of these studies use mammals as models, increasing numbers of studies are focusing on lipids in insects.

Lipids are detected in many tissues in insects, which include the midgut, ovaries, and imaginal discs; however, the important tissue for fat storage is the fat body (FB), which is the center of lipid metabolism (Toprak et al., 2020). The amount of lipids in insects varies widely and is affected by many factors including developmental stage, nutritional status, sex, environmental temperature, reproductive time and migratory flight. In general, female insects contain more fat than males, most likely including some fat stores for egg production (Toprak and Musselman, 2021). The lipid metabolism starts in the midgut, after food intake. The lipid compounds are first digested by lipases, with the products being transported by lipophorins to the targets, including the FB, ovaries and muscles. From here, fatty acids are transported to target cells by and fatty acid transport and binding proteins.

The lipids comprise a chemically diverse group of fatty acids, glycerolipids, glycerophospholipids, sphingolipids, sterols and prenols that perform many different functions in insects, both in physiological and pathological processes. Lipids are the main energy reserve material for many processes, such as embryogenesis, growth, development, metamorphosis, diapause, reproduction and prolonged flight, and are crucial in overwintering and enabling survival during periods of food shortage (Ryan and van der Horst, 2000). Significant quantities of neutral lipid are deposited in the developing oocyte during oogenesis, and in most species, the predominant fraction is triglyceride (Fruttero et al., 2017). In holometabolic insects, the body lipid content increases steadily during larval development, although not proportionally across larval stages; in fact, 95% of the energy for metamorphosis is derived from fatty acid oxidation (Palm and Rodenfels, 2020). In migrating insects, during intensive lipogenesis, carbohydrates from the diet are converted into lipids and stored in the fat body in the form of triglycerides (Hou et al., 2021). The epicuticular lipid layer is the main barrier preventing water loss in insects, thus allowing them to inhabit different environments (Wang et al., 2016).

**FIGURE 1:**
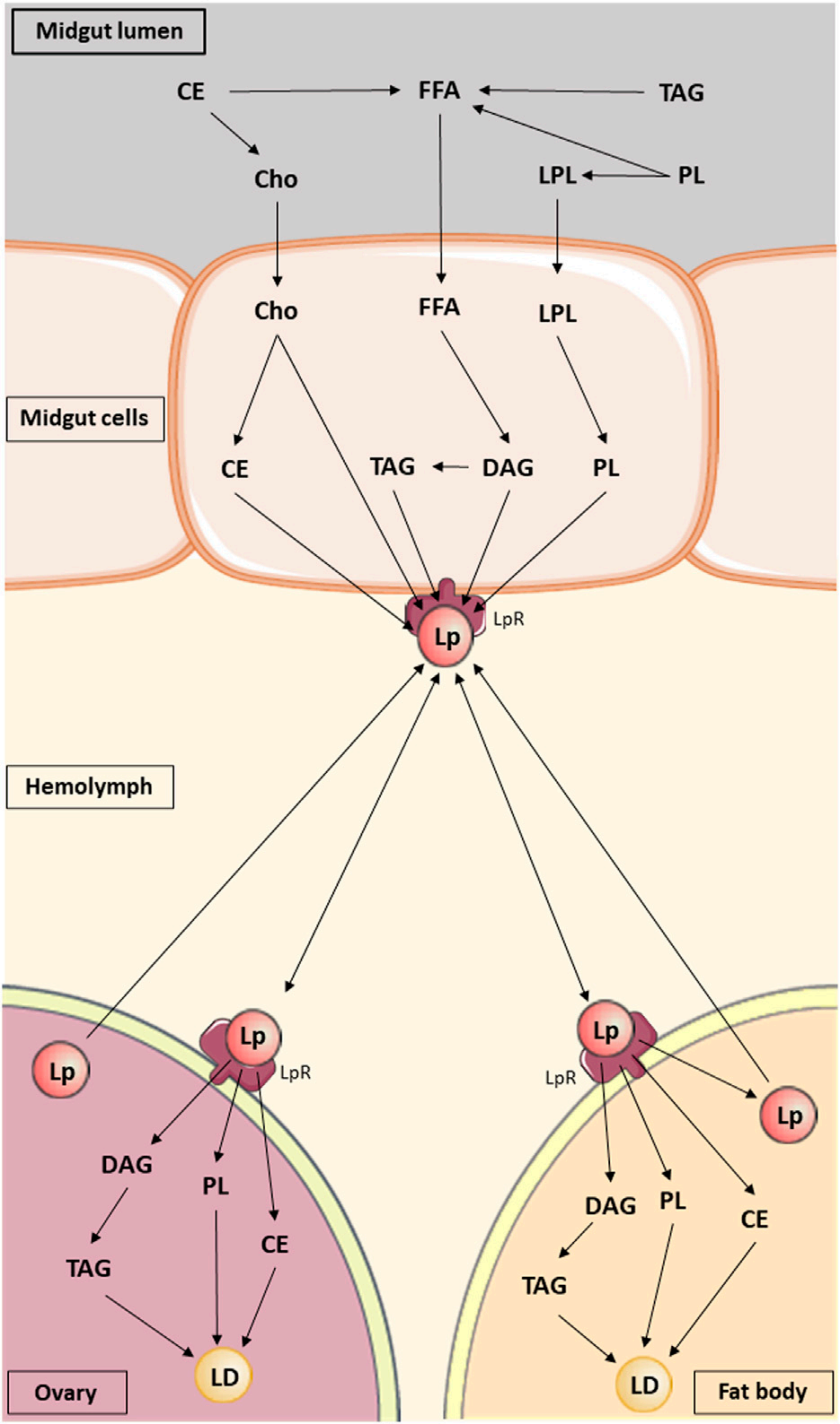
Lipid metabolism in insects. Lipids are digested at the midgut lumen and then absorbed and metabolized by midgut cells. Subsequently, they are transported in the hemolymph by lipophorin to fat body and oocytes, where they are stored in lipid droplets. Abbreviations: CE, Cholesteryl ester; Cho, Cholesterol; DAG, diacylglycerol; FFA, Free fatty acid; LD, Lipid droplet; Lp, Lipophorin; LPL, Lysophospholipid; LpR, Lipophorin receptor; PL, Phospholipid; TAG, triacylglycerol (based on the information in Majerowicz and Gondim (2013); structural formulas of chemical compounds from PubChem database).

Lipid synthesis, accumulation and hydrolysis takes place in the FB and triacylglycerol (TAG) accumulation predominantly occurs in intracellular lipid droplets (LDs) in adipocytes, the main cells in the FB (Skowronek et al., 2021). These processes are facilitated by fatty acid synthase (FAS) and perilipins, under the control of the insect endocrine system (Gondim et al., 2018; Toprak et al., 2020). The insect lipid metabolism scheme is given in Figure 1.

**FIGURE 2:**
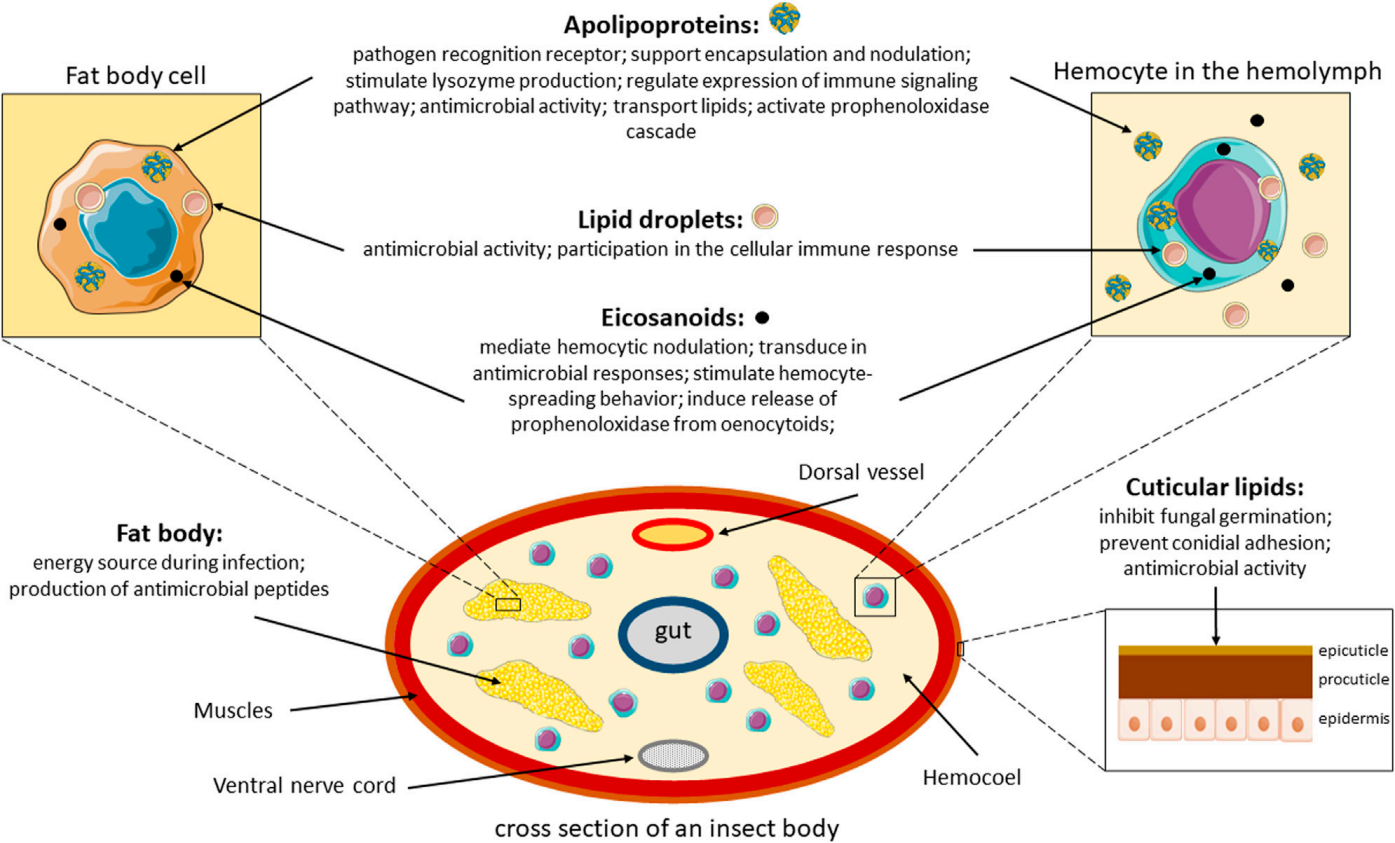
Overview of the role of lipids during infection in insects. Parts of the figure were drawn by using pictures from Servier Medical Art. Servier Medical Art by Servier is licensed under a Creative Commons Attribution 3.0 Unported License.

Lipids also play a key role in pathological processes, one of which is fungal infection. This review article describes how lipids are involved in the defense system of insects during infection with a particular focus on entomopathogenic fungal infections, as summarized in Figure 2. The review focuses on the protective functions of lipids as components of the cuticle and their participation in the cellular and humoral immune response. An important element of the work is also to discuss the role of lipid metabolites, such as eicosanoids, and the process of lipid peroxidation as a stress/inflammatory marker.

## 2 Cuticle–the first defense mechanism

In insects, the cuticular integuments are regarded as the first line of defense and the most important barrier protecting them against fungal infections (Butt et al., 2016; Balabanidou et al., 2018; Pedrini, 2018). Insect cuticles consist of an integument saturated with chitin, comprising a single-layered epithelium containing waxes, fatty acids and sterols. Moreover, the tracheas and the anterior and posterior intestines are also lined with chitin (Tajiri, 2017). The process of fungal adhesion occurs through three successive stages: firstly, adhesion of the fungal propagules to the surface of the cuticle took place; after this, the bond between the pregerminated propagules and the epicuticle is consolidated; finally, the fungi germinate and then develop on the cuticle itself. Following this, penetration structures such as the appressorium, penetration pegs or hyphae develop. Sclerotization and high content of chitin in the cuticle increase its protective potential in insects; in addition to preventing desiccation, some epicuticular lipids also have antifungal properties (Pedrini et al., 2007). Nevertheless, by adhering to the epidermis and penetrating the tissues of the host, fungal spores are still able to break through this protective barrier. Fungal spores produce a variety of cuticle-degrading proteases, lipases and chitinolytic enzymes (Samuels and Paterson, 1995).

Epicuticular lipids play an important role in preventing lethal desiccation, affecting insecticide and chemical penetration (Juarez, 1994). The epicuticle is hydrophobic, which is widely regarded as being suitable for fungal spore adhesion. However, some exceptions have been noted; for example, the booklouse, *Liposcelis bostrychophila* L., expresses fatty amides in the cuticule, which seem to prevent adhesion of (dry) conidia by entomopathogenic fungi (Lord and Howard, 2004). Although strategies based on preventing cuticular adhesion are rare, the hydrocarbon content of the waxy layer is known to influence fungal pathogenesis and a number of antimicrobial compounds are observed on the cuticle. For example, the cuticle of the Southern stink bug, *Nezara viridula* L. presents lipids and aldehydes that have a fungistatic effect on *Metarhizium anisopliae*, and *Heliothis zea* Boddie cuticle extracts are toxic towards *Beauveria bassiana* (Smith and Grula, 1982; Sosa-Gomez et al., 1997). In addition, a number of free fatty acids from Lepidoptera spp. and fatty acids from *Forcipomyia nigra* (biting midge) inhibited germination in many entomopathogenic fungi (Urbanek et al., 2012).

A combination of enzymatic and mechanical mechanisms facilitates penetration of the host cuticle. Entomopathogenic fungi are capable of producing various enzymes that break down the cuticle, including endoproteases, aminopetidases, carboxypetidases, N-acetylglucosaminidases, chitinases, esterases and lipases (Gillespie et al., 2000). It has been found that the cuticle-degrading ability of *C. coronatus* is related to its production of hydrolytic enzymes and the concentrations of certain compounds in the cuticles of susceptible and resistant insect species: some compounds are used by the fungus as nutrients, indicated by positive correlations, while others appear to be engaged in insect resistance, indicated by negative correlations (Bogus et al., 2017b; Wronska et al., 2018b).

*Conidiobolus coronatus*-resistant flies (*Calliphora vicina*, *Calliphora vomitoria*, *Sarcophaga carnaria, Musca domestica, Sarcophaga argyrostoma*) possess a cuticular lipid layer with a range of cuticle fatty acids, fatty alcohols, esters and sterols, together with tocopherol acetate and squalene, which are known to inhibit various activities of *C. coronatus* in a concentration-dependent manner (Golebiowski et al., 2008; Bogus et al., 2010; Golebiowski et al., 2011; Golebiowski et al., 2012b; Golebiowski et al., 2013a; Golebiowski et al., 2013b; Golebiowski et al., 2014a; Golebiowski et al., 2014b; Kaczmarek et al., 2020a; Kaczmarek et al., 2020b; Wloka et al., 2022).

*C. coronatus* conidia were unable to germinate on the cuticle of exposed *C. vicina* larvae, suggesting the presence of inhibitory compounds (Bogus et al., 2007). Moreover, *C. vicina* was found to be resistant to *C. coronatus* enzymes secreted into the culture medium *in vitro*, while *G. mellonella* and *Dendrolimus pini* were found to be susceptible; this host-specific resistance based on cuticle composition (Bogus et al., 2007; Bogus et al., 2017b; Wronska et al., 2018b). In fact, the three species have significantly different cuticle compositions: C14:0, C16:1 and C20:0 are present in *C. vicina*, while they are absent from *D. pini* and exist in trace amounts in *G. mellonella* (Golebiowski et al., 2008). FFAs C14:0, 16:0, 16:1, 18:0, 18:1, 18:2, 18:3, 20:0, and 20:1 inhibit *C. coronatus in vitro*, manifested as reduced sporulation, lower hyphal biomass, reduced ability to infect *G. mellonella* larvae and the release of less toxic products into the culture (Bogus et al., 2010); hence, these fatty acids may influence resistance to fungal attack.

The three insect species mentioned above employ different defense strategies upon contact with the pathogenic fungus, providing an effective physical and chemical barrier. In *C. vicina*, resistance to the fungus may be explained by the cuticle demonstrating high resistance to hydrolytic enzymes, as well as the possible presence of fungistatic compounds that can inhibit conidial germination, and its surface topography. Thanks to this strong protection provided by the cuticle, *C. vicina* can afford to reduce investment in defense mechanisms inside the hemocoel. Thus, the evolution of the cuticle in *C. vicina* may have been driven with the aim of reducing the overall cost of cellular and humoral defense: an ideal solution is a tradeoff between efficiency and cost.

### 2.1 Antimicrobial features of cuticular lipids

The cuticle lipids are involved in various types of chemical communication between insect species. They reduce the penetration of insecticides, chemicals and toxins, and provide protection against the attack of microorganisms, parasites, insects and predators. However, in addition to participating in the creation of a mechanical barrier, a key role played by cuticle lipids in protecting against infection is by offering antimicrobial properties (Rajabi et al., 2017).

The antimicrobial activity of free fatty acids (FFAs) has been described most widely in the literature. Their antimicrobial activity is influenced by length of the carbon chain, the nature of any double bonds that may be present, and by the presence of a hydroxyl group. In addition, unsaturated FFAs are more active than saturated FFAs (Zheng et al., 2005). Not all the mechanisms of the antibacterial action of fatty acids are fully understood, but the main mechanisms may be focused on the action on the cell membrane (Batalha et al., 2020).

The FFA mixture from *F. nigra* (containing C14:0, C16:1, C16:0, C18:2, C18:1, and C18:0) was found to be effective against bacteria, especially *Bacillus cereus* and *Enterococcus faecalis*; however, C9:0, C10:0, and C16:1 demonstrated the greatest activity (Urbanek et al., 2012). *Lucilla sericata* larva FFA extract was found to be effective against *Staphylococcus aureus* and *Streptococcus pneumoniae* and demonstrated significant anti-biofilm activity against the species (Liu et al., 2021). The minimum inhibitory concentration (MIC) of the cuticular FFA extract from *Rhynchophorus palmarum* was found to range between 1.5 and 20 μg/mL against Gram-positive bacteria (*Staphylococcus epidermidis, E. faecalis*), Gram-negative (*Pseudomonas aeruginosa, Escherichia coli, Klebsiella pneumonia*), and fungal species (*Candida albicans, Candida tropicalis*) (Batalha et al., 2020).

The cuticular fatty acids from *C. vomitoria*, *C. vicina*, *S. carnaria* and *L. sericata* was have also been found to demonstrate antifungal activity against entomopathogenic fungi (Golebiowski et al., 2012a; Golebiowski et al., 2013a; Golebiowski et al., 2013b; Golebiowski et al., 2014b). The *Galleria mellonella* FFAs C14:0, 16:0, 16:1, 18:0, 18:1, 18:2, 18:3, 20:0, and 20:1 displayed fungistatic activity against *C. coronatus in vitro*; the fungus demonstrated lower hyphal biomass, reduced sporulation, lower virulence against wax moth larvae and less toxic metabolites (Bogus et al., 2010). Gołębiowski et al. demonstrated the antifungal activity of cuticle lipids, including fatty alcohols (C10–C30) and various other compounds, e.g., butyl oleate, squalene and tocopherol acetate, from four flies: *C. vicina, C. vomitoria, M. domestica* and *S. carnaria*. Fatty alcohols C10–C30 have also exhibited moderate activity against fungal entomopathogens (*B. bassiana, Lecanicillium lecanii, M. anisopliae, Paecilomyces fumosoroseus, P. lilacinus*) and have been found to be highly effective against fungal pathogens affecting humans, such as *Aspergillus niger, C. albicans, C. lipolytica* and *C. tropicalis*. In addition, the compounds tocopherol acetate, butyl stearate and glycerol oleate, present in the cuticle, demonstrated moderate antifungal activity in both groups of fungal pathogens (Golebiowski et al., 2012b; Golebiowski et al., 2013b; Golebiowski et al., 2014a; Golebiowski et al., 2015).

## 3 Immunological system of insect

The second line of defense against pathogen infection is the innate immune system. Insects employ cellular and humoral defenses depending on the type of threat. In some insects, for example, *G. mellonella*, there is a strong structural and functional similarity of their immune system to the innate immune response described in mammals. Despite not being able to produce specific antibodies, insects are still able to produce various antimicrobial peptides (AMPs), which are then secreted to the hemolymph. While the humoral immune response acts through melanization, clotting and ROS production, cellular immunity acts through phagocytosis, nodulation and encapsulation (Dolezal et al., 2019; Ali Mohammadie Kojour et al., 2020; Ahlawat and Sharma, 2022).

### 3.1 The cellular response

The cellular response starts working immediately after the entry of the pathogen, and it is mediated by hemocytes, these being immunocompetent cells. The cellular response involves three main mechanisms of action: phagocytosis, nodulation and encapsulation (Sheehan et al., 2018). Insect hemocytes and mammalian neutrophils share many structural and functional features. (Bergin et al., 2005; Renwick et al., 2007; Browne et al., 2013). The pathogen is recognized by receptors on the surface of immune cells or by opsonins, proteins which are found in the hemolymph and bind to antigens. Recognition of the pathogen leads to the reorganization of the cytoskeleton of the hemocytes, thanks to which the cells spread to the pathogen penetration site (Marmaras and Lampropoulou, 2009; Zhang W. et al., 2021; Eleftherianos et al., 2021).

Hemocyte subpopulations may differ from one insect species to another. The *G. mellonella* larvae, the most popular insect model, includes five types of hemocytes: prohemocytes, plasmocytes, granulocytes, spherulocytes and oenocytoids (Senior and Titball, 2020). The majority of hemocytes circulating in the hemolymph are granulocytes and plasmatocytes, these being the only types capable of adherence (Hillyer, 2016). After adhesion to various surfaces, the circular plasmatocytes develop pseudopodia ranging in size from 10 to 15 µm wide and 20–30 µm long. Granulocytes are spherical, with a small nucleus and numerous granules in the cytoplasm. Upon encountering a foreign body, the granules undergo exocytosis, releasing compounds that act as opsonins and attractants for other hemocytes (Jiravanichpaisal et al., 2006; Lapointe et al., 2012). Spherulocytes and oenocytoids are non-adherent hemocytes. Spherulocytes transport cuticle components and contain numerous round and oval spheres that are bound to the cell membrane. Oenocytoids are round or oval cells with a small nucleus; these support melanization by carrying components of the phenoloxidase system. Oenocytoids can also release nucleic acids, which can warn about infection (Altincicek et al., 2008).

Four major types of hemocytes are described in *Drosophila*: prohemocytes, plasmatocytes, crystal cells, and lamellocytes. Plasmatocytes have similar function and morphology as vertebrate macrophages, while crystal cells and lamellocytes have similar functions to coagulation and granuloma formation in mammals (Tattikota et al., 2020). Lamellocytes are able to encapsulate foreign bodies such as parasitoid wasp eggs in *Drosophila* larvae, which cannot be phagocytosed. Finally, crystal cells enclose prophenoloxidase (ProPO), the most important enzyme in melanin biosysnthesis, which is stored as crystalline inclusions and released upon cell rupture (Lu and St Leger, 2016).

The total numbers and types of hemocytes are influenced by the developmental stage and environment, such as diet and stress factors (Vogelweith et al., 2016; Arteaga Blanco et al., 2017; Boguś et al., 2018). When the cellular response mechanisms are activated, the total hemocyte count (THC) and differential hemocyte count (DHC) change. Hemocytes neutralize foreign bodies by encapsulating them in capsules or nodules; interestingly, the spatial structure of nodules and capsules remains similar despite the contents (Ratcliffe and Gagen, 1976; Ratcliffe and Gagen, 1977). These structures may also contain melanin depending on the species (Hillyer, 2016).

Fungal infection induces several changes in cellular defense. The cellular defense mechanism was found to activate in *G. mellonella* in response to immunization with α-1,3-glucan from *A. niger*, reflected in changes in THC and DHC value, the formation of hemocyte aggregates and changes in apolipophorin III localization (Staczek et al., 2020). *Conidiobolus coronatus* infection results in damage to the *G. mellonella* hemocytes in several ways: by destruction of the cytoskeleton, more specifically the actin fibers (Kazek et al., 2020), resulting in cell death of hemocytes (apoptosis, autophagia) (Kazek et al., 2020; Wronska et al., 2021), oxidative stress (Kazek et al., 2020), and the production of highly toxic metabolites: coronatin-1, coronatin-2, dodecanol, octanoic acid, harman and norharman (β-carboline alkaloids) and two trichothecenes (HT-2 and T-2 toxic) (Wieloch et al., 2011; Paszkiewicz et al., 2016; Bogus et al., 2017a; Wronska et al., 2018a; Kazek et al., 2021; Kaczmarek et al., 2022). A significant decrease in the number of hemocytes was discoverd in *Spodoptera litura* treated with *Aspergillus flavus* compared to control. The decline may be due to cytotoxic fungal activities, which inhibit the activity of defensive hemocytes to support the fungal infection (Karthi et al., 2018). Similar results were presented by Kaur et al. , who report a decrease in *S. litura* hemocyte counts after treatment with the fungus *Alternaria alternata* (Kaur et al., 2015).

In locusts, the cellular defense is moderated by hemocytes and hematopoietic tissue. In *Locusta migratoria*, hemocytes and hematopoietic tissue work together to clear invading pathogens from the hemocoel. A β-1,3-glucan infection of *L. migratoria* induces nodule formation, increases apoptosis in hematopoietic tissue, resulting in a considerable loss of hemocytes in circulation and a consequent instant increase of hemocytes and hematopoietic tissue cells to support the host cellular defense (Duressa et al., 2015). It is believed that the key phagocytes protecting against invasion by *Metarhizium acridum* in *L. migratoria* are plasmatocytes and granulocytes. During fungal infection, an increase of amount of locust hemocytes was observed in the initial days after infection, as well as a decrease in the next period of infection compared to the control group (Cao et al., 2016; Yu et al., 2016).

### 3.2 The humoral response

The cellular immune response is assisted by the mechanisms of the humoral response. Antigen recognition by PRRs (pattern recognition receptors) activates two main signaling cascades: the Toll and Imd (immune deficiency) pathways (Wang X. et al., 2019; Lin et al., 2020). Their activity induces the transcription of genes encoding pathogen-specific effector proteins, including antimicrobial peptides (AMPs) (Wu et al., 2018). Enzymatic cascades also regulate the process of coagulation and melanization, as well as the production of ROS and RNS (Clark, 2020).

Toll receptors were first discovered while studying the dorsal-ventral (DV) polarization of *Drosophila melanogaster* embryo (Nusslein-Volhard and Wieschaus, 1980). Extensive phylogenetic analyses have confirmed the presence of the Toll pathway in many organisms from protozoa to mammals (Brennan and Gilmore, 2018). This pathway is triggered in response to infections by Gram-positive bacteria or fungi; it also influences the genes associated with antibacterial and antifungal proteins (Shin et al., 2006; Geng et al., 2021). The ligand for Toll receptors is Spatzle, also known as Spaetzle or Spätzle (Spz). Its activation results in the Toll receptor - Spatzle complex being cleaved by the serine protease SPE (Spaetzle processing enzyme) (Rahimi et al., 2019; Zheng et al., 2020). The attachment of Spz to the transmembrane Toll receptor, found on the surface of the cells of the fat body, activates the intracellular signaling cascade. It includes, among others, the adapter proteins MyD88 and Tube, and Pelle kinase. After activation, they phosphorylate the Cactus protein (homologous to the Iκ-B protein), which exists as a complex with the transcription factors Dif and Dorsal; this phosphorylation contributes to the release of transcription factors and their subsequent transfer to the cell nucleus (Carvalho et al., 2014; Chowdhury et al., 2019; Sarvari et al., 2020; Zhang K. et al., 2021). Following this, the genes encoding AMP are transcribed (Yu et al., 2020).

The peptidoglycan of Gram-negative bacteria is characterized by the existence of meso-diaminopimelic acid (PGN-Dap). This molecular pattern is attached by the PRGP-LC (peptidoglycan recognition protein LC) receptor on the surface of the cells of the fat body (Liu et al., 2020); PRGP-LC is the first protein in the Imd pathway. Its dimerization gives rise to a signal cascade (Wang Q. et al., 2019), resulting in the attaching of the Imd protein homologous to the RIP (receptor-interacting protein) in mammals. The dFADD kinase is then bound to the Dredd caspase, which is homologous to caspase 8 in mammals. This complex activates the kinases TAK1 (TGF-beta activated kinase 1) and MAPK, which in turn transfer the signal to the IKK (IκB kinase) complex, which includes Ird5 and Kenny. The complex cleaves the N-terminal part of the Relish protein (Goto et al., 2018). The active form of this protein enters the cell nucleus, where it regulates the transcription of genes encoding antimicrobial peptides (De Gregorio et al., 2002).

Literature data indicates that both the Toll and Imd pathways have the potential to be initialized in *L. migratoria* in response to *M. acridum* infection, and that Toll/IMD genes demonstrate different expression in the locust fat body and hemocytes (Zhang et al., 2015). Moreover, the locust Toll pathway can be activated before fungal penetration as a result of fungus β-1,3-glucan detection (Zheng et al., 2020). *Drosophila* Toll pathway mutants were found to be more susceptible to infection by *B. bassiana* and *M. anisopliae* (entomopathogenic fungi) or by the opportunistic pathogen *Aspergillus fumigatus* compared to wild type flies (Lemaitre et al., 1997; Rutschmann et al., 2000; Tauszig-Delamasure et al., 2002)*.* In addition, infection studies with *B. bassiana* showed that Persephone’s protease was very important for Toll receptor activation in response to fungal infection (Levitin and Whiteway, 2008). Mutant GNBP-3 defective *Drosophila* flies were unable to effectively induce drosomycin expression following challenge with fungal cell wall components such as β1-3 glucans, as well as heat-killed *C. albicans* cells and *Aspergillus nidulans* cell extracts (Gottar et al., 2006). The lethal effect, influenced in the Toll pathway, was observed also after injection into *Drosophila* human pathogens, like *A. fumigatus*, *C. albicans* and *Cryptococcus neoformans* (Lemaitre et al., 1996; Alarco et al., 2004; Apidianakis et al., 2004). In *S. exigua*, infection with *B. bassiana* and *Metarhizium rileyi* activated the eicosanoid biosynthesis via the Toll signal pathway, but not Imd (Park and Kim, 2012a; Roy and Kim, 2022).

One of the best understood signal transduction cascades is the JAK-STAT pathway. Upon infection, the extracellular cytokine Unpaired (Upd) binds to the cellular Domeless (Dome) receptor, thus activating the Jak/Stat pathway. Domeless is phosphorylated by Hopscotch (Hop). This recruits Stat, which is then dimerized and translocated to the nucleus. This results in the activation of antimicrobial gene transcription such as nitric oxide synthase (Trivedi and Starz-Gaiano, 2018; Moore et al., 2020). The JAK/STAT pathway is crucial during viral infection, but the molecular mechanisms observed in *Bombyx mori* and *Aedes aegypti* following challenge with *B. bassiana* suggests that it may have a supplemental role in antifungal response (Geng et al., 2016). Differences in JAK-STAT gene expression have also been detected in Colorado potato beetles during *Metarhizium robertsii* infection (Kryukov et al., 2021).

## 4 Role of lipids in the insect immune mechanisms

One of the regulating factors of the insect immune system are lipids, that enter the organism from the environment (e.g., with food) and those produced by the body through various pathways. For example, feeding on a lipid diet (linseed oil) increased mortality in *Manduca sexta* after *Serratia marcescens* infection (Adamo et al., 2007), and the peanut oil, which contains mostly TAGs, induced melanin formation in *B. mori* (Li et al., 2022). Although the effect of lipids on various elements of the immune response of mammals is well understood and widely described in the literature, their effects on insects are less studied. Most research has described the effect of arachidonic acid (ARA) application. For example, 4% ARA, supplied with the diet, reduced mortality in honeybees *Apis mellifera* infected with *E. coli* and increased phenoloxidase, antitrypsin, and lysozyme acticvity; they also elevated the mRNA expression of the defensin-2, toll, myd88, and dorsal genes associated with the immune system (Yu et al., 2021). ARA injection significantly increased the humoral and cellular immune responses in *Spodoptera exigua* (Hasan et al., 2019) and increased adhesion of *G. mellonella* hemocytes without significant effect on cytoplasmic cAMP levels, suggesting that the ARA may stimulate an alternative non-cAMP pathway of adhesion in the wax moth (Marin et al., 2005). ARA injection reverses the inhibition of *Rhodnius prolixus* hemocyte phagocytosis caused by *Trypanosoma rangeli* infection (Figueiredo et al., 2008) and induces the expression of the cecropin and lysozyme genes in *B. mori* (Morishima et al., 1997). 10-Hydroxy-2-decenoic acid (10-HDA), a fatty acid present in high amounts in royal jelly, inhibits the secretion of extracellular polymeric substances, reducing the adhesion and aggregation of *S. aureus* and disrupting biofilm architecture (Gao et al., 2022). Octanoic acid, a short fatty acid, deforms hemocytes, disorders networking, activates apoptosis and/or necrosis, activates caspases 1–9 and elevates the 8-hydroxy-2' -deoxyguanosine (8-OHdG) level in *G. mellonella* hemocytes after *in vivo* and *in vitro* application (Kaczmarek et al., 2022). Compared to flies reared on a standard diet, *Drosophila* reared on diets with higher cholesterol, Wolbachia strains exhibited lower pathogen blockage and 2- to 5-day earlier viral-induced mortality (Caragata et al., 2013). Peanut triacylglycerol injection induced melanin formation in *B. mori* (Li et al., 2022).

**FIGURE 3:**
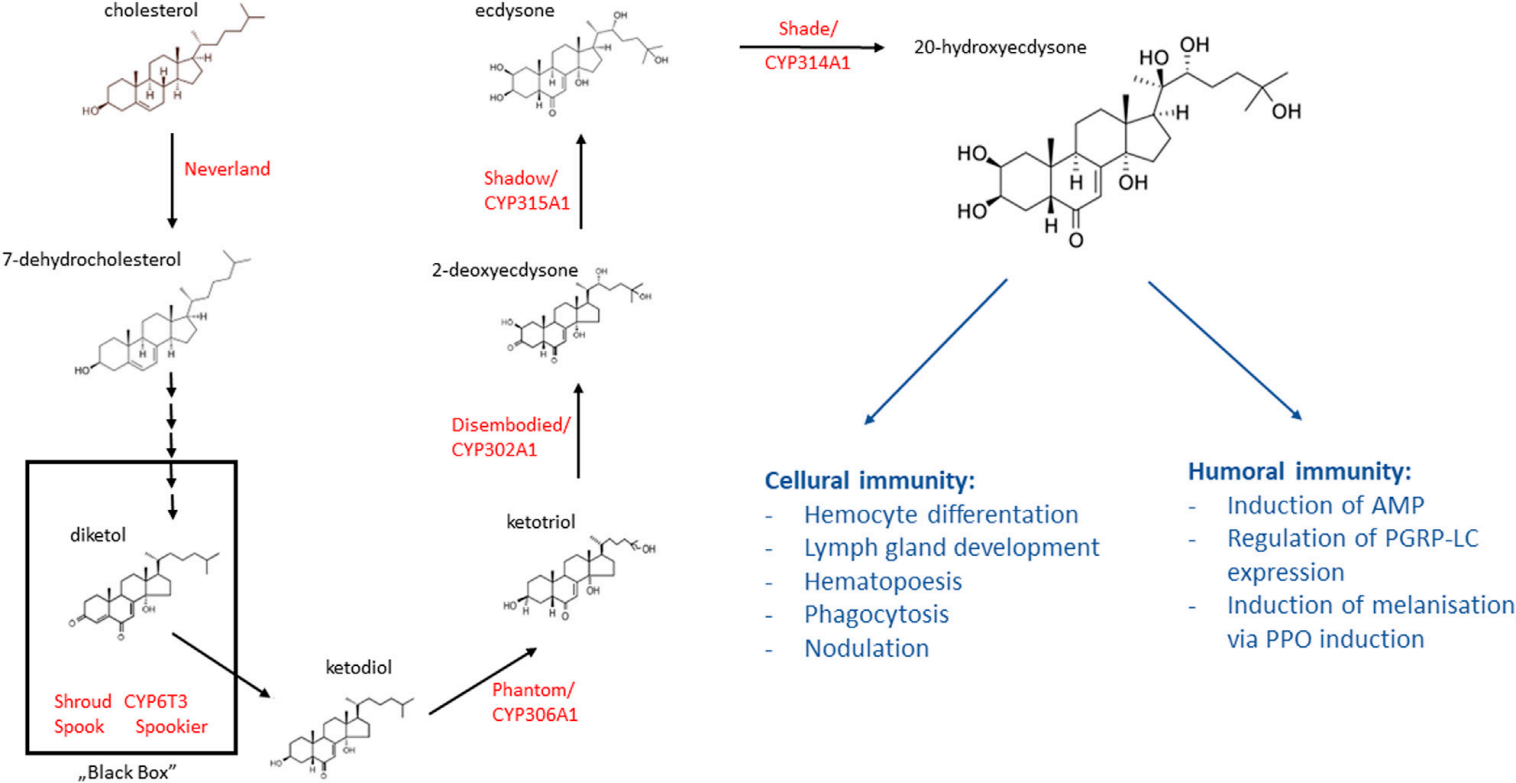
The ecdysteroid biosynthesis pathway in the prothoracic gland and the role of 20 dehydroecdysone in insect immunity. Cholesterol is converted into 20-dehydroecdysone (20E) by several ecdysteroidogenic enzymes (red font). Ecdysteroid biosynthesis starts from the conversion of cholesterol to 7-dehydrocholesterol (7 dC). Then, 7 dC is converted to ketodiol via multiple steps with the participation of a group of enzymes called “Black Box.” In the terminal catalytic steps of ecdysteroid biosynthesis, ketodiol is then sequentially hydroxylated at carbon 25, carbon 22, carbon 2, and lastly carbon 20, resulting in a conversion to the active steroid hormone 20E (based on the information in Niwa and Niwa (2014), Saito et al. (2016), Chanchay et al. (2019); structural formulas of chemical compounds from PubChem database).

A supply of cholesterol is needed in the diet to maintain the immune system during infection, as this cannot be synthesized by insects. This compound is a precursor of steroid hormones, of which ecdysone is the most important for the immune system. Ecdysone (also called α-ecdysone) is produced in the prothoracic gland in the form of a prohormone with low biological activity, from which the active form 20E (also called β-ecdysone) is biosynthesized in peripheral tissues. The 20E biosynthetic pathway in insects is shown in Figure 3. As ecdysone secretion is primarily responsible for determining the timing of moulting, it has been classified as a “moulting hormone.” However, developmental profiling of 20E responses indicate that the hormone also regulates other physiological processes, including those associated with metabolism, stress response and immunity. In *D. melanogaster*, 20E is involved in increasing the phagocytic activity of hemocytes and lymph gland development and hematopoiesis. In the flesh fly, *Neobellieria bullata*, nodulation is stimulated by laminarin, a β-1,3-glucan major component of fungal cell walls; this process is conjugated with the level of eicosanoids, the metabolites of some polyunsaturated fatty acids, and is stimulated by 20E (Franssens et al., 2006). In addition, 20E has been found to regulate the PPO (prophenoloxidase) system to resist fungal (*M. anisopliae*) invasion by modulating the pattern recognition receptor GNBP-2 in the global pest *L. migratoria* (Han et al., 2020).

Some entomopathogenic fungi have developed mechanisms to target ecdysone as an infection facilitator. *Beauveria bassiana* appears to produce ecdysteroid 22-oxidase (MrE22O), which inactivates ecdysone in *G. mellonella*. MrE22O deletion results in impaired virulence compared to the wild-type strain; the hosts demonstrated increases in both ecdysone and antimicrobial gene expression (Zhu et al., 2021). 20-Hydroxyecdysone binds to the nuclear ecdysone receptor (EcR), affecting gene transcription. EcR knockdown significantly inhibited locust mortality when infected with *M. anisopliae* (Han et al., 2020).

## 5 The role of the fat body in immunity

In the insect, the fat body plays an analogous role to the adipose tissue and liver in vertebrates. It plays a key role in regulating various processes, such as larval growth, immunology and courtship behavior, by regulating hormone and nutrition signals and that influence the brain (Arrese and Soulages, 2010).

The fat body undergoes significant changes in the insect life cycle, undergoing changes in form in the embryonic stage, growth in the larval stage and remodeling in the pupal stages; it also affects adult reproduction, a process regulated by hormones and nutrients, including lipids. The fat body is made up of five main types of cells (trophocytes, oenocytes, mycetocytes, chromatocytes, and urocytes) which vary in composition, size, and function during the different stages of growth (Skowronek et al., 2021).

In adipocytes, lipids are mainly stored as triacylglycerols (TAG), which are mobilized by the adipokinetic hormone depending on the current metabolic needs of the body. TAGs store energy for the flying muscles and egg cells, and are also used during the functioning of the immune system. The exact mechanisms that cause lipid mobilization during infection and the effect of mobilized lipids are not yet known; however, it is known that lipids can be a source of energy and/or for membrane biogenesis at sites of infection or in hemocytes (Arrese and Soulages, 2010). The immune signaling activation shifts anabolic lipid metabolism from triglyceride storage to phospholipid synthesis to support immune function in the fat body. The activation of *Drosophila* larvae fat body Toll signaling leads to a tissue-autonomous reduction in triglyceride storage which is paralleled by decreased transcript levels of the DGAT homolog *midway*, which carries out the final step of triglyceride synthesis. In contrast, Kennedy pathway enzymes that synthesize membrane phospholipids are induced (Martinez et al., 2020).

A considerable increase in fatty acid methyl ester content was noted in the fat body of *Zophobas morio* larvae on the fifth day of *Metarhizium flavoviride* infection; this level then decreased on day seven. However, the levels of other esters, especially octanoic acid decyl ester/OADE, were lower days 5 and 7 after infection (Golebiowski et al., 2020). Experiments on mosquitoes, *Ae. aegypti*, infected with Gram (+) bacteria and fungi found a significant increase in the expression of two fat body genes involved in lipid metabolism, a lipid carrier protein lipophorin (Lp) and its lipophorin receptor (LpRfb); however, no such increase was noted for Gram (−) bacteria. In the fat body, immune induction caused by pathogen and parasite infections found both Lp and LpRfb gene expression to be regulated by the Toll/REL1 pathway (Cheon et al., 2006).

The chronic activation of the IMD/NF-κB pathway in *Drosophila* prevents gut bacteria-dependent sterol regulatory element binding protein (SREBP) processing and thus lipid metabolism. By restricting the diffusion of PGN to the fly hemolymph, the PGRP-LB^sec^ enzyme allows gut bacteria-dependent lipogenesis in remote adipocytes and promote fly survival. In the absence of such brake, lipid storages of orally infected flies are rapidly depleted and life span is reduced (Charroux and Royet, 2022). When bacteria such as *Mycobacterium marinum* are injected into *Drosophila* body cavity, the transcription factor Mef2, which activates transcription of metabolic genes in non-infected individuals, switches its activity to enhance transcription of immune genes. As a result, anabolic transcripts are reduced and energy stores, such as lipids, are lost. Toll and the IMD signaling pathways are acting genetically upstream of Mef2 in this process (Clark et al., 2013).

The production of antimicrobial peptides (AMPs) can be considered as the main role of the fat body in the immune response in insects. Both the innate humoral (innate level of immune proteins, e.g., lysozyme) and acquired immune responses induce the synthesis of antibacterial attacin and defensin proteins. After contact with a pathogen, chemokines stimulate the production of immune proteins and polypeptides in the fat body; these relay information between the hemolymph and the fat body. In addition, effectors are produced a few hours after body cavity infection and these are then transferred to the hemolymph (Salcedo-Porras et al., 2022).

Antimicrobial peptides are produced in the fat body by the activity of the Toll and Imd pathways. AMP expression is also regulated by ecdysone, a hormone produced as a result of biochemical transformation of cholesterol (Zanarotti et al., 2009; Zheng et al., 2018; Huang et al., 2020; Kenney et al., 2020; Nunes et al., 2021). Based on their structure and aa sequence, AMPs can be classified into three groups: 1) cecropins—linear peptides with an α-helix but lacking cysteine residues; 2) defensins—with six to eight conserved cysteine residues, a stabilizing system of three or four disulfide bridges, together with three other domains with a flexible loop at the amino terminus; and 3) peptides—overrepresented with proline or glycine residues (Makarova et al., 2018). The seven families of these proteins identified in *Drosophila* (attacins, cecropins, defensin, diptericins, drosocin, drosomycins, and swordfish) have been most widely described (Lazzaro et al., 2020). However, 18 such proteins have been characterized in *G. mellonella*, five of which have been extensively studied (cecropins, gallerimycin, galliomycin, morcin-like protein and gloverin-like protein) (Sheehan et al., 2018).

Most AMP proteins are antibacterial, but there are also some with antifungal properties, such as drozomycin from *D. melanogaster*, helomycin from *Heliothis virescen*s, thermicin from *Pseudocanthothermes spiniger*, gallerimycin from *G. mellonella* (Hegedus and Marx, 2013) and AP2 (anionic peptide 2) from *G. mellonella* (Sowa-Jasilek et al., 2020). The AMPs are probably involved in the initial recognition and destruction of fungal structures in the outer layer of the cuticle (Butt et al., 2016). *B. mori* cecropin A and gloverin showed high antifungal activity against the entomopathogenic fungus *B. bassiana* in both *in vitro* and *in vivo* research studies (Lu et al., 2016). The infection of *Anopheles stephensi* with *B. bassiana*, an entomopathogen which produces oosporein, can downregulate the expression of antifungal peptide genes in the fat body (Feng et al., 2015; Wei et al., 2017). Infection of *G. mellonella* larvae by the entomopathogenic *B. bassiana* caused a time-dependent increase in the expression of gallerimycin and defensin (galiomicin) at the mRNA level, but only weak, transient expression of the gene for cecropin ((Wojda et al., 2009). On the other hand, injection of filamentous fungus *Fusarium oxysporum* resulted in elevated defensin, proline-rich peptide 2, cecropin D-like peptide and anionic peptide 1 productoin in *G. mellonella* (Mak et al., 2010). Infection of *D. melanogaster* with the *B. bassiana* or *M. anisopliae* (fungi) resulted in elevated gene expression for the antifungal peptide drosomycin and metchnikowin; however, no increase was observed for the antibacterial peptides diptericin or cecropin A. Various fungi can also stimulate the antimicrobial peptide attacin A through the Imd and Toll pathways, thus activating the transcription factors Relish and/or Dif, as observed in the *Dif* and *Relish* mutants of *D. melanogaster*. Interestingly, while attacin A expression required Relish in response to *Geotrichum candidum* but Dif in response to *B. bassiana*, indicating that the immune system can distinguish between the two species (Hedengren-Olcott et al., 2004). Moreover, *D. melanogaster* hemolymph demonstrated a number of changes in protein expression following exposure to yeast *Saccharomyces cerevisiae*, as indicated by proteomic analysis (Vierstraete et al., 2004).

## 6 Lipid droplets and insect immunity

Lipid droplets (LDs) are found in almost all organisms from bacteria to humans, and a wide range of cell types, including those involved in the mammalian immune response. These specialized lipid-storing organelles comprise a hydrophobic core with high amounts of neutral lipids, e.g., triglyceride, cholesteryl ester or retinyl ester, surrounded by a phospholipid or cholesterol monolayer. It is also accompanied by a range of associated proteins which play roles in cell homeostasis, metabolism and signaling (Melo et al., 2011; Olzmann and Carvalho, 2019; Lundquist et al., 2020). In insects, these organelles are found mainly in the cells of the fat body, mainly the oenocytes (Wei et al., 2019), in the intestine (gut) (Kuhnlein, 2012) and in hemocytes (Shin et al., 2020).

In mammals, LDs are ubiquitous organelles that modulate immune and inflammatory responses. There are also literature reports about the role of these organelles in the insect immune response. Due to the diverse interactions between pathogens and LDs, and the strong evolutionary pressure, LDs are key mediators of the immune system. *Drosophila* LDs were found to sequester histones via the Jabba receptor (Li et al., 2012) and release them in response to bacterial infection (Anand et al., 2012).

*Ae. aegypti* Aag2 immune response cells were found to accumulate LDs following exposure to *Enterobacter cloacae*, Sindbis and Dengue viruses. The LDs also accumulated in cells in the midgut following *S. marcescens* and Sindbis virus challenge or after a blood meal. These LD numbers were also increased by constitutive activation of Toll and IMD pathways following knockdown of Cactus and Caspar: their respective negative modulators (Barletta et al., 2016). The fatty acids released as a result of triglyceride breakdown are thought to have antibacterial properties similar to detergents, weakening bacterial viability by disrupting their membranes (Brasaemle et al., 2004).

In *Drosophila*, the LDs in the fat body exhibit different morphological dynamics between transient and sustained bacterial infection. More specifically, *perilipin1 (plin1)*, a core gene regulating LDs metabolism, is suppressed by the IMD pathway via the Martik (MRT)/Putzig (PZG) complex. During transient activation, *plin1* is reduced, resulting in the production of large LDs, thus alleviating the oxidative stress caused by ROS production during immune reactions (Wang et al., 2021). Research of Harsh and co-workers pointed that *Drosophila* LDs might play role of inflammation markers, and can dictate the outcome of the infection, depending on the nature of the challenge. Authors pointed that the systemic infection of *Drosophila* adult flies with non-pathogenic *E. coli*, the extracellular bacterial pathogen *Photorhabdus luminescens* or the facultative intracellular pathogen *Photorhabdus asymbiotica* results in intestinal steatosis marked by lipid accumulation in the midgut. Accumulation of LDs in the midgut also correlates with increased whole-body lipid levels characterized by increased expression of genes regulating lipogenesis. The lipid-enriched midgut further displays reduced expression of the enteroendocrine-secreted hormone, Tachykinin. The observed lipid accumulation requires the Gram-negative cell wall pattern recognition molecule, PGRP-LC, but not PGRP-LE, for the humoral immune response (Harsh et al., 2019). LDs are also involved in the social immunity of insects. In the social aphid *Nipponaphis monzeni*, the soldier nymphs possess large numbers of highly-differentiated large hemocytes in the body cavity; these contain considerable amounts of LDs and phenoloxidase (PO). In contrast, the hemolymph accumulates large amounts of tyrosine and a unique repeat-containing protein (RCP). The insects live in a nest made from plants, a *gal.* Upon attack, the soldier nymphs erupt from the gall and discharge fluid containing LDs from their large hemocytes; they mix this fluid with their legs, and use it to close the hold in the gall by forming a lipidic clot. At the same time, the activated PO converts tyrosine to reactive quinones; these link RCP and other macromolecules together, thus reinforcing the clot and sealing the breach (Kutsukake et al., 2019).

## 7 Fatty acid metabolites—eicosanoids—in insect immunity

**FIGURE 4:**
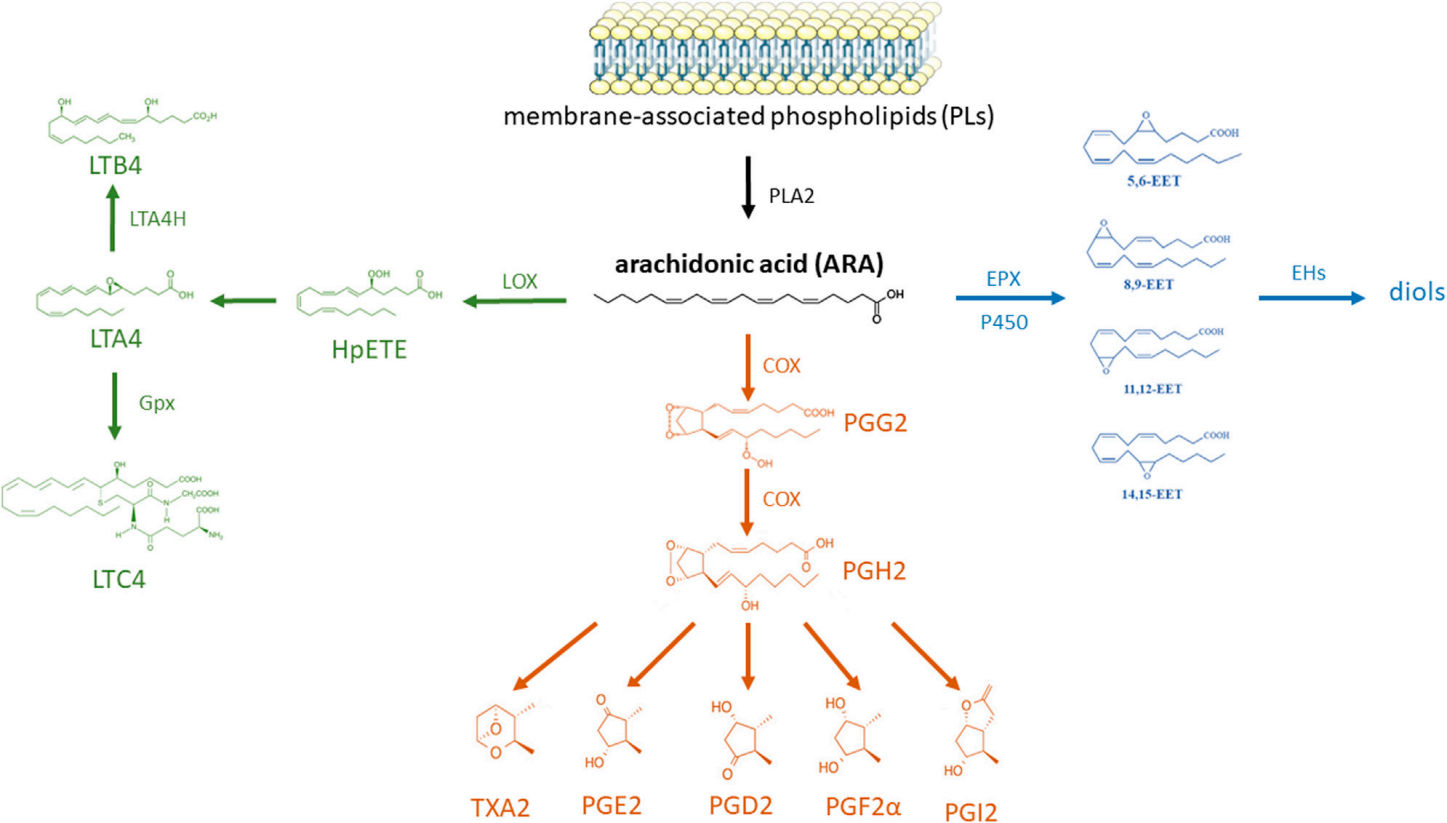
Eicosanoid biosynthesis and degradation in insects. Phospholipase A2 (PLA2) catalyzes the hydrolysis of linoleic acid (LA) from membrane-associated phospholipids (PLs); the resulting LA is elongated by long-chain fatty acid elongase (Elo) and then desaturated by desaturase (Des) to arachidonic acid (ARA). ARA is then oxygenated by epoxidase (EPX) into epoxyeicosatrienoic acid (EET), lipoxygenase (LOX) into leukotriene (LT), or cyclooxygenase-like peroxynectin (Pxt) to prostaglandin (PG). The EETs are degraded by soluble epoxide hydrolase (sEH). LTA4 is formed from 5-hydroxyperoxide eicosatetraenoic acid (HpETE) and changed into LTB4 by LTA4 hydrolase (LTA4H) or into LTC4 by glutathione peroxidase (Gpx). Finally, various PGs are formed from PGH2 by cell-specific enzymes, thromboxane A2 (TXA2) synthase (TXAS), PGD2 synthase (PGDS), PGE2 synthase (PGES), and PGI2 synthase (PGIS); these PGs are degraded by PG dehydrogenase (PGDH) and PG reductase (based on the information in Kim and Stanley (2021); structural formulas of chemical compounds from PubChem database; part of the figure were drawn by using pictures from Servier Medical Art. Servier Medical Art by Servier is licensed under a Creative Commons Attribution 3.0 Unported License).

In insects, lipids act on the immune system not only directly, but also through their metabolites. The main group of metabolites with immunomodulatory activity are eicosanoids and their biosynthesis is well described in insects. The pathways (Kim and Stanley, 2021) for the synthesis of eicosanoid groups from arachidonic acid are shown in Figure 4.

Eicosanoids mediate hemocyte nodulation reactions to fungal infections (Stanley, 2006). They have been found to be involved in signal transduction in antimicrobial responses in *M. sexta* after infection by the entomopathogenic fungi *B. bassiana* and *M. anisopliae* (Dean et al., 2002; Lord et al., 2002)*.* The *M. rileyi* infection of beet armyworm, *S. exigua,* caused the activation of PLA2 and phenoloxidase (PO) enzymes; moreover, the increase of expression by genes encoding cecropin, gallerimycin, and hemolin, the specific antimicrobial peptides was detected (Roy and Kim, 2022). *S. exigua* PLA2 activation was also detected in the fat body and hemocytes in response to infection of other entomopathogen, *B. bassiana* (Park and Kim, 2012b). Infection with the entomopathogenic fungus *C. coronatus* results also in increased PLA2 activity in *G. mellonella* hemolymph (Wronska et al., 2021). Eicosanoids such as prostaglandin E2 and 17- hydroxyeicosatetraenoic acid, appear to be downregulated in *B. mori* after *B. bassiana* infection. Prostaglandin E2 (PGE2) mediates the cellular immune response by stimulating the spread of hemocytes, thus enabling phagocytosis, nodulation and encapsulation (Groen et al., 2012; Ahmed and Kim, 2021; Roy et al., 2021). PGs also induce the release of prophenoloxidase (PPO) from oenocytoids into the hemolymph; PPO is then converted to the active form, phenoloxidase (PO) to enable the formation of melanin around any nodules and capsules (Shrestha and Kim, 2008). Furthermore, studies on *S. exigua* larvae found that injection of eoxyoctadecamonoenoic acids (EpOMEs), vernolic acid (12,13-EpOME) and coronaric acid (9,10-EpOME) suppressed the cellular immune responses following exposure to bacteria; In addition, EpOME treatment also suppressed AMP gene the expression (Vatanparast et al., 2020). PGs also reduced ovarian development of *R. prolixus* after injection of the non-entomopathogenic fungus *A. niger* into the hemocoel (Medeiros et al., 2009). Also, formosan subterranean termites, *Coptotermes formosanus*, demonstrated significantly increased mortality after treatment with *Isaria fumosorosea* and ibuprofen sodium salt (eicosanoid biosynthesis inhibitor) compared to insects treated with fungus alone or untreated insects.

Although arachidonic acid (ARA) is a precursor to the synthesis of eicosanoids, its concentration in the body of insects is kept at a low level. Stanley and Kim propose that terrestrial insects limit ARA levels to minimize oxidative stress. Compared to other animals, they have developed a relatively high metabolic rate and a highly-developed tracheal system that directly supplies the correct amount of oxygen needed for the proper functioning of active tissues, such as flight muscles; this may make them more susceptible to reactive oxygen species (ROS) generated by high oxidative catabolism. Long-chain PUFAs, including ARA, react with ROS and undergo peroxidation, which in turn leads to various forms of cellular damage (Stanley and Kim, 2020).

## 8 Apolipoproteins

Apolipoproteins (Apo) act as carrier proteins; they bind lipids to form called lipophorins, a form of lipoprotein particle. In insects, Apo constitute the main components of insect lipophorin particles, being referred to as apolipophorins (ApoLp). Lipophorin (Lp) is present in the hemolymph of various insects, where it shuttles lipids such as diacylglycerol, phospholipids, sterols and hydrocarbons between tissues (Ryan and van der Horst, 2000). Insect lipophorin comprises two non-exchangeable apolipophorins, apolipophorin I (ApoLp-I, ∼240 kDa) and apolipophorin II (ApoLp-II, ∼80 kDa), as well as apolipophorin III, an exchangeable protein (ApoLp-III, ∼18 kDa). ApoLp-I and ApoLp-II are formed from the common precursor apolipophorin II/I (ApoLp-II/I) by post-translational cleavage. ApoLp-II/I is homologous to the mammalian apoB and ApoLp-III to mammalian apoE (Van der Horst and Rodenburg, 2010).

ApoLp-III plays an important role in the innate immune system, where it appears to modulate the insect cellular response. It is considered to be a pathogen recognition receptor (PRR) because it binds microbial cell wall components, such as lipopolysaccharide (LPS) in Gram-negative bacteria, lipoteichoic acids (LTA) in Gram-positive bacteria, and β-1,3-glucan in fungi, thus stimulating phagocytosis and nodulation by insect hemocytes (Whitten et al., 2004; Maravilla et al., 2020). In *G. mellonella*, ApoLp-III supports the phagocytosis of yeast cells by insect plasmatocytes: it affects the adherent properties of hemocytes and, after binding with lipids, enhances the process of encapsulation and nodulation (Gotz et al., 1997; Zakarian et al., 2002; Whitten et al., 2004). ApoLp-III exerts its antibacterial properties by *inter alia* stimulating lysozyme production, and increasing its concentration in the hemolymph (Zdybicka-Barabas et al., 2013). In addition to directly inhibiting *B. bassiana*, silkworm *B. mori* apolipophorin-III (BmApoLp-III) also influences various genes related to the Toll and Jak/STAT immune signaling pathways; it also promotes immune effector expression, and indirectly inhibits *B. bassiana* reproduction (Wu et al., 2021). ApoLP-III has been found to be an LTA neutralizing protein, since in *G. mellonella*, ApoLp-III was found to bind to LTAs produced by *B. subtilis*, thus preventing the loss of plasmatocytes, suggesting that it may protect the insect against the toxin (Halwani et al., 2000). Apolipophorin III is an antibacterial agent against human pathogens (Fallon et al., 2011). The growth of *Legionella dumoffii* can be inhibited by ApoLp-III from *G. mellonella* (Staczek et al., 2018). On the other hand, injection of *E. coli, Bacillus thuringiensis or B. bassiana* induces ApoLp-III expression in *Apis cerana* fat body (Kim and Jin, 2015). Gupta et al. also confirm that ApoLp-III appears to participate in the midgut immune defense of *Anopheles gambiae* against *Plasmodium berghei* (Gupta et al., 2010).

The influence of apolipoproteins on the immune system of insects is related not only to antimicrobial activity. ApoLp-III may also initiate the formation of low-density lipophorins (LDLp), in response to infection; these are then taken up by granulocytes, thus signalling infection to hemocytes (Dettloff et al., 2001; Niere et al., 2001). Significant induction of ApoLp-III expression, correlating with a strong nodulation response, was noted in both six- and eight-instar *Thitarodes pui* larvae compared with saline-injected controls, 1 hour after *B. bassiana* conidial challenge (Sun et al., 2012). In addition, *G. mellonella* plasmatocytes and granulocyte subpopulations displayed impaired adhesion to glass slides after ApoLp-III treatment *in vitro* and after injection of ApoLp-III into the hemocoel of larvae. The authors also postulate that ApoLp-III may downregulate nodule formation and/or phagocytosis (Zakarian et al., 2002). In contrast, Whitten et al. (2004) report greater nodule formation *in vivo* in *G. mellonella* larvae after injection of ApoLp-III, which may suggest that ApoLp-III has a stimulating role in the cellular response. Proteomic analysis showed that together with other apolipoproteins, ApoLp-III was a component of *G. mellonella* net-like coagulation structures, which also include endogenous extracellular nucleic acids. Furthermore, ApoLp-III was found to be a specific RNA-binding protein, indicating that it may play a role in the extracellular RNA-mediated immune response (Altincicek et al., 2008). In addition, apolipophorin I and II (ApoLp-II/I) are involved in the transport and deposition of surface-cuticular lipids in *L. migratoria* (Zhao et al., 2020), and in locusts, ApoLp-III is one of the elements activating the prophenoxidase cascade, which is a critical step in the immune response in defense against pathogens (Mullen and Goldsworthy, 2003).

In *Gryllus texensis*, immune function was found to be dependent on the amount of free ApoLp-III in the hemolymph: a decrease in the amount of free ApoLp-III in the hemolymph resulted in an impaired immune response due to the action of adipokinetic hormone. In addition, increasing post-flight ApoLp-III levels by injecting purified ApoLp-III also reduced flight-induced immunosuppression (Adamo et al., 2008). *G. mellonella* hemolymph samples collected from larvae after injection with ApoLp-III demonstrated strongly increased antibacterial activities against *E. coli* as well as clearly enhanced lysozyme-like activities (Niere et al., 1999). Also ApoLp-III lowered the adhesion of *B. subtilis* to wax moth hemocytes and reduced the ability to remove bacteria from the hemolymph (Zakarian et al., 2002).

Although most research indicates that ApoLp-III may have a role in the antifungal defense mechanism, some data suggests that apolipoproteins I/II (ApoLp-II/I) may also have an important role (Wang X. et al., 2019). ApoLp-II/I and ApoB are homologues, and the latter plays a key role in innate immunity in mammals. Kamareddine et al. found that silencing *Apo-II/I* modulates the immune response by increasing resistance to bacterial (*E. coli*) and fungal (*B. bassiana*) infections in *A. gambiae* in a TEP1 (thioester 1-containing protein) dependent manner, *via* the JNK pathway (Kamareddine et al., 2016). Enhanced TEP1-dependent resistance to *Plasmodium* infection was observed in Apo-II/I knockout mosquitoes, suggesting that Apo-I and II are key components the immunological system in this insect. (Rono et al., 2010). *B. mori* ApoLp-II/I is capable of binding *S. aureus via* lipoteichoic acids on the cell surface and by repressing hemolysin gene expression (Hanada et al., 2011; Omae et al., 2013). A recent study on ApoLp-II/I in *Antheraea pernyi* found this protein to have a negative role in prophenoloxidase activation (Wen et al., 2017).

## 9 Lipid peroxidation as a stress/inflammatory marker

An imbalance between ROS (reactive oxygen species) and antioxidant levels contributes to oxidative stress. ROS arise naturally from metabolic activity and during an immune response, and can cause damage to lipids, proteins and DNA, (Azzi, 2022). One of the most widely-studied physiologically significant effects of ROS is lipid peroxidation; the process induces cellular injury and is an accurate indicator of oxidative stress. The resulting lipid peroxides are unstable, and decompose to form various compounds via initiation, propagation and termination processes (Leuti et al., 2019; Bahja and Dymond, 2021). In the cell membrane, ROS primarily induce lipid peroxidation by their reacting with polyunsaturated fatty acids (Wen et al., 2017).

In insects, lipid oxidation is correlated with the inflammatory processes that occur during infection. The entomopathogenic fungi *I. fumosoroseus* and *Hirsutella thompsonaii* are capable of causing mortality in cockroaches (*Periplaneta americana*) by inducing oxidative stress (Chaurasia et al., 2016) *C. coronatus* infection also induced oxidative stress, autophagy and apoptosis response in *G. mellonella* (Kazek et al., 2020; Wronska et al., 2021). The significant change in the level of lipid peroxidation was detected in *Dysdercus koenigii* hemocytes after *A. niger* infection (Kumar et al., 2019). Another fungus, *A. flavus* induced oxidative stress and immunosuppressive activity in *S. litura* (Kaur et al., 2021), and the entomopathogenic *B bassiana* was found to kill the rice-striped stem borer, *Chilo suppressalis*, by causing lipid peroxidation (Shamakhi et al., 2020). In mosquito larvae (*Aedes caspius*), *Bacillus thuringiensis Kurstaki* (Btk) infection induced significantly higher levels of two key markers of oxidative stress: lipid peroxidation and protein oxidation. In imago mosquitoes, significantly higher lipid peroxidation compared to controls was observed 12 and 24 h after Btk inoculation, but only after 12 h following *E. coli* inoculation (Ahmed, 2011). Herrera-Ortiz et al. report that H_2_O_2_ treatment appears to activate the anti-malarial immune response in a malaria-refractory strain of *A. gambiae* following a meal on infected blood, compared to a susceptible strain (Herrera-Ortiz et al., 2011).

Malpigian tubules of *Pectobacterium carotovora* infected *Drosphila* excrete hemolymphatic lipids mediated by a stress-induced lipid binding protein, materazzi. Lack of materazzi results in raised hemolymph levels of reactive oxygen species (ROS) and elevated lipid peroxidation in flies. It is speculated that such excretion of hemolymphatic lipids is a physiological adaptation protecting host tissues from excessive ROS response to microbial infection (Li et al., 2020). Suppression of humoral and cellular immune responses of *B. mori* invaded by the dipteran endoparasitoid *Exorista bombycis* is accompanied by increased H_2_O_2_ concentration in hemocytes, cytotoxicity, lipid peroxidation and membrane porosity (Pooja et al., 2017).

The lipid oxidation process is also indirectly related to the immune response. Works on the *Drosophila* lymph gland performing the function of hematopoietic organ, have provided evidence that fatty acid oxidation (FAO) is indispensable for the differentiation of hemocyte progenitors as in the absence of FAO, the progenitors were unable to differentiate, and exhibited altered histone acetylation, while supplementation of acetate restored these defects. Jun-Kinase (JNK), involved in the differentiation of hemocyte progenitors, regulates transcription of CPT1/whd (withered), the rate-limiting enzyme of FAO. For the proper functioning of the insect immune system the proper differentiation and maturation of hemocytes is decisive (Tiwari et al., 2020).
